# Metabolic features of tumor-derived extracellular vesicles: challenges and opportunities

**DOI:** 10.20517/evcna.2024.12

**Published:** 2024-08-27

**Authors:** Pilar Espiau-Romera, Andrés Gordo-Ortiz, Inés Ortiz-de-Solórzano, Patricia Sancho

**Affiliations:** Hospital Universitario Miguel Servet, IIS Aragón, Zaragoza 50009, Spain.

**Keywords:** Extracellular vesicles, cancer, metabolism, metabolites, biomarkers

## Abstract

Tumor-derived extracellular vesicles (TDEVs) play crucial roles in intercellular communication both in the local tumor microenvironment and systemically, facilitating tumor progression and metastatic spread. They carry a variety of molecules with bioactive properties, such as nucleic acids, proteins and metabolites, that trigger different signaling processes in receptor cells and induce, among other downstream effects, metabolic reprogramming. Interestingly, the cargo of TDEVs also reflects the metabolic status of the producing cells in a time- and context-dependent manner, providing information on the functionality and state of those cells. For these reasons, together with their ability to be detected in diverse biofluids, there is increasing interest in the study of TDEVs, particularly their metabolic cargo, as diagnostic and prognostic tools in cancer management. This review presents a compilation of metabolism-related molecules (enzymes and metabolites) described in cancer extracellular vesicles (EVs) with potential use as cancer biomarkers, and discusses the challenges arising in this rapidly evolving field.

## INTRODUCTION: FUNCTION, CLASSIFICATION AND MARKERS

The term extracellular vesicles (EVs) refers to a heterogeneous population of membrane-derived vesicles secreted by both eukaryotic and prokaryotic cells. In the early days, EVs were considered only a cellular mechanism to dispose of unwanted material, but owing to advances in purification techniques and subsequent molecular analyses, we currently know that EVs are involved in many different crucial physiological functions such as stem cell maintenance, tissue repair, immune surveillance, and blood coagulation^[1-3]^. In this sense, EVs play a major role in cellular and organismal communication and signaling, since they transmit information to distant cells by activating surface receptors or transferring intracellular contents, including biomolecules such as proteins, lipids, nucleic acids and sugars, via internalization^[4]^. Considering that the cargo (content) of EVs mirrors the phenotype of the secreting cells and their relative abundance in biofluids, the analysis of EVs from liquid biopsies has great diagnostic potential for different diseases, such as cancer^[5]^. In fact, they have been detected in most body fluids including blood, urine, saliva, breast milk, cerebrospinal fluid, semen, amniotic fluid, breath condensate, and ascites^[6-11]^.

EVs were initially classified into three subtypes on the basis of their mechanism of biogenesis: exosomes, microvesicles, and apoptotic bodies. While exosomes (40-150 nm) are exclusively formed through the endolysosomal pathway, microvesicles (150-1,000 nm) arise from budding at the plasma membrane. On the other hand, apoptotic bodies (1-5 μm) are formed only during programmed cell death^[12]^. In 2018 and 2023, the International Society for Extracellular Vesicles (ISEV) redefined EVs as “particles released from the cells that are delimited by a lipid bilayer and cannot replicate”^[13,14]^. Considering the difficulties in reaching a global consensus in the classification of EVs and the absence of specific markers for each subtype, the ISEV still recommends the use of operational terms for EV classification, but with caution. For example, EVs with a diameter smaller than 200 nm are called S-EVs (small), and those with a larger size should be considered L-EVs (large)^[15]^. However, these cut-offs are not strict and separation methods such as ultracentrifugation yield populations of mixed sizes. In this sense, we have adapted the nomenclature used in papers cited in this review to adhere as much as possible to the current classification.

EVs express several common surface molecules that have been consistently used in isolation and characterization techniques, such as CD9, CD81, CD63, CD82, flotillin, TSG101, Alix, and heat shock proteins (HSP60, HSP70, HSPA5, and CCT2)^[16]^. Among these markers, tetraspanins such as CD9, CD63, or CD81 are the most commonly used since they are ubiquitously expressed across cell types and their expression is especially high^[17]^. The family of tetraspanins shares a common structure consisting of four transmembrane domains enriched in polar residues followed by an extracellular loop and conserved cysteines on the extracellular side. This configuration enables tetraspanins to interact with other family members through homophilic interactions, as well as with receptors and signaling molecules at the membrane through heterophilic interactions, thus creating a complex pleiotropic signaling network that regulates EV biogenesis, cargo and uptake^[18]^.

## TUMOR-DERIVED EXTRACELLULAR VESICLES

The study of tumor-derived extracellular vesicles (TDEVs) has attracted great attention in the past few years, considering their exciting potential for diagnostic purposes in liquid biopsies, especially in cases where direct regular biopsy is not always possible^[19]^. Interestingly, while the release of EVs is relatively universal across cell populations, cancer cells tend to produce more vesicles overall, facilitating their detection in biofluids where EVs originating from other normal cells and tissues are present^[20]^. Although TDEVs express the general EV markers mentioned in the previous section (several examples are included in Supplementary Table 1), the expression of cancer-specific markers has also been described (a selection of markers is shown in Table 1), especially for diagnostic and therapeutic purposes. Indeed, immunoaffinity-based isolation techniques using cancer-specific markers represent the only way to enrich or even purify TDEVs in complex biological samples, as described previously^[32]^.

**Table 1:** A selection of cancer-specific protein markers found in TDEVs

**Biomarker**	**Source of EVs**	**Cancer type**	**Application**
CD147^[21]^	Serum	Colorectal cancer	Diagnosis and prognosis
EGFRvIII^[22]^	Plasma	Glioblastoma	Prognosis
EpCAM^[23]^	Plasma	Colorectal cancer	Early diagnosis
Glypican-1^[24]^	Serum	Pancreatic cancer	Diagnosis
HSP70^[25]^	Plasma	Melanoma	Prognosis
LGALS3BP^[26]^	Serum	Endometrial cancer	Early diagnosis
LRG1^[27]^	Plasma	Non-small cell lung cancer	Diagnosis
MDA-9, GRP78^[28]^	Serum	Metastatic melanoma	Early diagnosis
PDCD6IP, FASN, XPO1, ENO1^[29]^	Plasma	Prostate cancer	Diagnosis
PD-L1^[30,31]^	Serum	Metastatic melanoma	Prognosis
TYRP2^[21]^	Plasma	Glioblastoma	Diagnosis
VLA-4^[25]^	Plasma	Melanoma	Diagnosis

Notably, some of these markers have also been described for additional cancer types. TDEVs: Tumor-derived extracellular vesicles; EVs: extracellular vesicles.

Interestingly, in addition to conventional targets such as cytosolic proteins or tetraspanins, different groups have highlighted the potential of detecting specific nucleic acids encapsulated within TDEVs for diagnosis^[33]^, including microRNAs (miRNAs), circular RNAs (circRNAs), or long noncoding RNAs (lncRNAs). Although beyond the scope of this literature review, we provide some examples in Supplementary Table 2.

The increased release of EVs by cancer cells underscores their pivotal role in tumor homeostasis and progression, mediating the active communication between the tumor microenvironment and the primary tumor. Thus, TDEVs contribute to multiple cancer hallmarks, including tumor initiation, angiogenesis, metabolic reprogramming, pluripotency, metastasis, and immunosuppression^[20]^. While the implications of TDEVs in these phenomena have been comprehensively reviewed elsewhere^[34,35]^, we provide some representative examples below.

During cancer initiation, the dynamic interplay between proliferating tumor cells and surrounding stromal cells is crucial for maintaining a proper microenvironment, which explains why cancer-associated fibroblast (CAF) activation is increased by TDEVs^[36,37]^. For example, S-EVs from bladder cancer cells carry cytokines such as transforming growth factor Beta (TGF-β), which triggers CAF differentiation via activation of the SMAD pathway^[38]^. On the other hand, miR-630 or miR-210 contained in ovarian and lung cancer TDEVs may prompt fibroblasts differentiation into CAFs via NF-kB and Janus kinase 2 signals, respectively^[39,40]^.

Interestingly, TDEVs allow horizontal information transfer between different tumor cell populations or even with nontransformed cells during the initiation and progression stages. In 2008, Al-Nedawi *et al*. first demonstrated that a heterogeneous population of vesicles derived from brain tumors enhanced the aggressiveness and proliferation of their less aggressive counterparts^[41,42]^. Subsequent studies revealed how the activated isoform of the epithelial growth factor receptor (EGFR), EGFRvIII - a major cargo of TDEVs - was horizontally transferred to other cancer cells within the TME^[41]^. Notably, pro-invasive proteins such as CD44 or CD155 are often associated with TDEVs expressing EGFRvIII in glioma cells^[43,44]^, suggesting that the coordinated transfer of several molecules is associated with aggressiveness. On the other hand, the driver oncogene K-Ras was enriched in TDEVs derived from mutant pancreatic cancer cells, thereby increasing the invasiveness and proliferation of nontransformed wild-type cells^[45,46]^.

Moreover, TDEVs seem to be crucial for the maintenance of cancer stem cells (CSCs), the main tumoral subpopulation contributing to chemoresistance and relapse^[47]^. The most frequently described mechanism for stemness enhancement mediated by TDEVs is the activation of the Wnt/Notch/β-catenin pathway in CSCs by biological components present in the cargo of vesicles released by either differentiated cancer cells^[48]^ or stromal cells such as CAFs^[49]^. On the other hand, CSCs have been proposed to reprogram non-CSCs through CD44v6-dependent RTK, GPCR, and integrin activation facilitated by their TDEVs in pancreatic cancer^[50]^.

As briefly mentioned above, communication via TDEVs facilitates metastasis by two different mechanisms. On the one hand, S-EVs produced by tumor cells or CAFs induce EMT in recipient cells by transferring miRNAs, which promote the upregulation of EMT markers such as vimentin, N-cadherin, ZEB1, SNAIL, SLUG, and TWIST1 and the activation of the AKT or ERK pathways in breast or prostate tumors^[51,52]^. On the other hand, TDEVs mediate the formation of the metastatic niche in specific target organs, such as prostate cancer spread to the bone^[53]^. Indeed, the identity and location of the recipient cells in target organs or tissues are finely regulated by TDEVs: Hoshino *et al*. reported that metastatic cancer cells that form S-EVs with a distinct signature of integrins not only promote tumor colonization in nonmetastatic tumors, but also alter and redirect the tissue to be colonized^[54]^.

Recent studies have indicated that TDEVs also play a role in immunosuppression via different mechanisms. First, checkpoint proteins such as PD-L1 have been identified in EVs from melanoma and brain cancer cells^[55,56]^. Indeed, Ricklefs *et al*. reported that patients suffering from an aggressive form of melanoma, but not healthy individuals, harbored TDEVs carrying PD-L1^[55]^. Consequently, when these TDEVs were injected *in vivo*, the number of CD8^+^ T cells was significantly reduced^[57]^. Moreover, the polarization of macrophages toward M2 pro-tumoral behavior could also be explained by S-EVs activating diverse signaling pathways such as the PI3K/AKT, STAT3, p38, MAPK, ERK or NF-κB pathways, which then induce the synthesis and secretion of immunosuppressive molecules and interleukins, such as IL-6, IL-8, IL-10, and arginase-1^[40,56]^. A third proposed mechanism involves the inhibition of immune cells by ATP hydrolysis mediated by the nucleotidases CD39 and CD73, which can be present in TDEVs produced by high-grade serous ovarian cancer^[58]^. These nucleotidases produce adenosine from ATP, which binds to the surface of recipient cells, triggering a signaling cascade that leads to the buildup of cytosolic cAMP and inhibition of cell functions^[59]^.

Finally, TDEVs contribute to cancer chemoresistance through diverse mechanisms, which involve mainly direct regulation of drug efflux and promotion of prosurvival signaling. Indeed, oral squamous cell carcinoma cells with both *de novo* and acquired resistance to cisplatin eliminate the drug by accumulating it inside S-EVs^[60]^. TDEVs can also transfer the ATP-binding cassette (ABC) transporter P-gp to chemosensitive cells^[35]^. On the other hand, miR-21 or miR-24 contained in TDEVs induce chemoresistance by targeting the tumor suppressor genes PDCD4 and PTEN, thus promoting cell survival in leukemia, breast cancer, and squamous cell carcinoma^[34,61]^. Moreover, S-EVs produced by breast cancer cells after treatment with paclitaxel are enriched in survivin, which promotes cell survival and resistance to apoptosis^[62]^.

## METABOLITE CARGO OF TDEVs

In a normal physiological state, the most efficient form of energy production for cells is based on mitochondrial oxidative phosphorylation, in which carbohydrates, especially glucose, as well as proteins and lipids, are catabolized into intermediates that enter the tricarboxylic acid (TCA) cycle^[63]^. In the context of cancer, cells undergo a series of profound changes in their bioenergetics, which are necessary to support rapid cell growth and proliferation in environments characterized by oxygen and nutrient scarcity. As a result, tumor cells undergo a process of metabolic reprogramming, where they promote nutrient uptake and activate anabolic biosynthetic pathways. The most common changes associated with metabolic reprogramming in cancer are increased glucose uptake and metabolism through glycolysis, elevated glutamine consumption, lipid and amino acid biosynthesis, and redox homeostasis^[64]^.

Although far less studied than nucleic acids and proteins, EVs carry numerous metabolites in their cargoes, which can reflect the actual metabolic state of the producing cells in a time- and context-specific manner. Considering the profound metabolic reprogramming suffered by cancer cells, it is not surprising that an increasing number of metabolites specifically up- or downregulated in TDEVs compared with noncancer tissues are being discovered in a variety of biofluids such as urine, blood or saliva^[65]^. In this sense, the analysis of the TDEVs metabolite cargo can be useful for cancer diagnosis or prognosis evaluation in the clinical setting, since the detection of cancer-specific metabolites in a complex biological sample containing EVs from different sources would be indicative of the presence of cancer cells.

In the following subsections, we summarize a selection of changes in the metabolic cargo of TDEVs reported in the literature in recent years, classified by metabolic pathway. When available, we also discuss the clinical implications of the findings.

### Glycolysis

Metabolic reprogramming in cancer has been classically associated with increased glucose consumption in tumor cells as a source of energy and building blocks necessary to meet their increased proliferative needs. Unlike normal cells, tumor cells exhibit high levels of glycolysis and reduced mitochondrial respiration, even in the presence of oxygen, leading to a state termed aerobic glycolysis or the Warburg effect^[66]^. In general, the glycolytic state is characterized by increased expression of glycolytic enzymes [e.g., hexokinase II (HK2), phosphofructokinase I (PFK1), lactate dehydrogenase (LDH) and pyruvate kinase II (PKM2), among others] and glucose and lactate transporters [glucose transporter 1 (GLUT1) and monocarboxylate transporters 1 and 4 (MCT1, MCT4)], as well as the accumulation of oncometabolites (lactate, glutamate, fumarate and succinate). In that sense, lactate can be found at high concentrations in S-EVs, especially under hypoxic conditions when glycolysis is further increased^[67]^. Interestingly, Joshi *et al.* reported higher levels of L-lactic acid in S-EVs from breast cancer patients with residual disease than in those with a complete response to neoadjuvant chemotherapy^[68]^. Moreover, glycolic acid, a byproduct of glycolysis, was also found to be one of the most important increased metabolites in cell-derived S-EVs from colorectal cancer patients^[69]^. These examples illustrate the advantage of metabolite detection in detecting TDEVs in complex clinical samples.

### Lipid metabolism

Cancer cells reprogram their lipid metabolism to sustain important cellular functions such as the synthesis of the cell membranes needed for increased proliferation, the biosynthesis of lipid-derived signaling molecules, and energy production^[70]^. Indeed, the plasma membrane of cancer cells shows important differences in lipid composition compared with that of normal cells, promoting changes in membrane fluidity and favoring cellular signaling through the formation of cholesterol-rich lipid rafts. In this sense, lipids are especially important in TDEVs not only because of their crucial function in EV biogenesis and membrane formation but also because of their ability to reflect the lipid composition of cancer cell membranes^[71]^. Indeed, the majority of studies characterizing lipid metabolism in TDEVs have identified differences in membrane structural lipids (glycerophospholipids, glycolipids, and cholesterol), some of which suggest their use as disease biomarkers, as summarized in Table 2.

**Table 2:** Metabolic markers for lipid metabolism found in TDEVs, arranged by tumor type

**Cancer type**	**Sample type**	**Metabolites**	**Application**	**Ref.**
Breast cancer	Plasma	↑ Lyso-phosphatidylcholine	Diagnosis, prognosis	[72]
	Plasma	↑ Diglycerides ↓ Cholesteryl esters	Diagnosis	[73]
Colorectal cancer	Supernatant cells	↑ Glycerolipids, cholesterol and sphingolipids	Diagnosis	[74]
	Supernatant cells and serum	↑ Glycerophospholipid, arachidonic acid and propionate	Diagnosis	[69]
	Feces	↑ Fatty acids	Diagnosis	[75]
	Plasma	↓ Phosphatidylcholine	Diagnosis	[76,77]
Hepatocellular carcinoma	Plasma	↑ Sphingosines, dilysocardiolipins, lyso- phosphatidylserine, 1-hydroxy fatty acids ↓ SM4, acylGlcSitosterol	Diagnosis	[78]
Lung cancer	Serum	↑ Cholesteryl esters ↓ Phosphatidylcholine	Diagnosis	[79]
Melanoma	Plasma positive CD81 EVs	↑ Fatty acids	Prognosis	[80]
Oesophageal squamous cell carcinoma	Plasma recurrent and nonrecurrent	↑ Palmitoleic acid, palmitaldehyde, isobutyl decanoate	Prognosis	[81]
Ovarian cancer	Supernatant cells	↑ GM3, zymosterol, cholesteryl esters, lysophosphatidic acids ↓ Ceramides, digalactosyl diglycerides, phosphatidic acids	Diagnosis	[82]
Pancreatic cancer	Serum	↑ Lyso- phosphatidylcholine, plasmenyl- phosphatidylcholine, phosphatidylethanolamine	Diagnosis	[83]
	Supernatant cells	↑ Diglycerides	Prognosis	[84]
Prostate cancer	Supernatant cells	↑ Phosphatidylserine, glycosphingolipids, sphingomyelin, cholesterol	Diagnosis	[85]
	Urine	↑ Steroids, steroid hormone dehydroepiandrosterone sulfate	Diagnosis	[86]
	Supernatant cells	↑ Cholesteryl esters	Diagnosis	[87]
Renal cell carcinoma	Urine	↑ Lysophospholipids, phosphatidylcholine, phosphatidylethanolamine and glycerolipids	Diagnosis	[88]

Note that the main text is organized by lipid species. The arrows indicate an increase (↑) or decrease (↓) in the concentration. TDEVs: Tumor-derived extracellular vesicles; EVs: extracellular vesicles.

For example, Buentzel *et al.* reported elevated levels of lyso-phosphatidylcholine in plasma L-EVs from breast cancer patients compared with those from controls and demonstrated their association with worse overall survival, suggesting that it is a potential diagnostic and prognostic biomarker^[72]^. Similarly, the levels of lyso-phosphatidylcholines and other phosphoglycerides such as plasmenyl- and phosphatidylethanolamine were found to be increased in S-EVs isolated from the serum of patients with pancreatic cancer. These lipids are correlated with the tumor markers CA19-9, CA242, and CEA and other clinical parameters such as tumor stage or overall survival, suggesting their potential value as pancreatic cancer biomarkers^[83]^. Indeed, detection of these lipids in EVs may improve the diagnostic and prognostic value of CA19-9, the most widely used clinical biomarker for PDAC diagnosis and patient follow-up.

Although a correlation with clinical parameters has not been reported, several studies have demonstrated an increased content of certain membrane lipids (among others) in TDEVs, reinforcing their potential as disease biomarkers. For example, structural phosphatidylserine and cholesterol, together with glycosphingolipids and sphingomyelin, have been shown to be abundant in EVs isolated by ultracentrifugation from prostate cancer cells^[85]^. A similar pattern has been described in colorectal cancer cells, where S-EVs from the LIM125 cell line have been reported to contain higher levels of glycerolipids, cholesterol, and sphingolipids than nontumoral controls^[74]^. Similarly, Eylem *et al.* reported that colorectal cancer S-EVs upregulate lipids via pathways related to the metabolism of glycerophospholipids, arachidonic acid, and propionate both in cell culture and in clinical samples^[69]^. In addition, other authors have demonstrated an alteration in fatty acids in fecal-derived EVs and a decrease in phosphatidylcholine in plasma-derived EVs from colorectal cancer patients^[75-77]^, all of which were isolated by ultracentrifugation and, thus, likely represent a heterogeneous population of EVs.

In addition, cholesteryl esters have been proposed as diagnostic and prognostic biomarkers in patient samples from several cancer types. For example, Smolarz *et al.* reported elevated levels of cholesteryl esters and lower levels of phosphatidylcholine in serum-derived S-EVs from lung cancer patients than in those from healthy controls^[79]^. Moreover, Brzozowski *et al.* detected a very high abundance of cholesteryl esters in S-EVs derived from metastatic prostate cancer cells, indicating increased accumulation in samples representing late disease/metastatic stages^[87]^. Similarly, Nishida-Aoki *et al*. reported that, compared with those from low-metastatic cells, plasma S-EVs from highly-metastatic breast cancer cells presented lower levels of cholesteryl esters and were more enriched in unsaturated diglycerides, which was strongly associated with the stimulation of angiogenesis^[73]^.

In contrast to the other structural lipids described above, some lipid species are important bioactive molecules that mediate cell signaling and communication in cancer and are upregulated in TDEVs. Indeed, Altadill *et al*. reported that EVs isolated by ultracentrifugation from PANC-1 cells treated with transforming growth factor beta have higher levels of diglycerides than those from control cells, suggesting a role in cell-to-cell communication and signaling^[84]^. Interestingly, TDEVs isolated from the sucrose gradient of supernatants from breast cancer cells contained PGE2, which is important for the induction of myeloid suppressor cells, thus promoting immune evasion^[89]^. Furthermore, some studies using prostate cancer urinary EVs (both S- and L-EVs) have demonstrated abnormal levels of steroids such as androgens and the steroid hormone dehydroepiandrosterone sulfate, suggesting a potential role of EVs in androgen signaling to neighboring cells during disease progression and perineural invasion^[86]^.

### Amino acids

Extensive changes in the levels of different amino acids and derivatives resulting from metabolic reprogramming have been reported in cancer. Interestingly, these changes can be detected not only in plasma from cancer patients but also in EVs from different body fluids^[65,90]^ [Table 3].

**Table 3:** Amino acids found in TDEVs

**Cancer type**	**Sample type**	**Metabolites/Enzymes**	**Application**	**Ref.**
Colorectal Cancer	Supernatant cells, serum, stool	↑ Lysine	Diagnosis	[69]
	Supernatant cells, serum	↑ Glycine, valine, tryptophan ↓ Proline	Diagnosis	[91]
	Supernatant cells	↑ Proline	Diagnosis	[92]
Lung cancer	Pleural fluid	↑ Leucine and phenylalanine	Diagnosis	[93]
Pancreatic cancer	Plasma patients treated with chemotherapy	↑ Alanyl-histidine, 6-dimethylaminopurine, leucyl-proline, methionine sulfoxide	Recurrence	[94]

The arrows indicate an increase (↑) or decrease (↓) in the concentration. TDEVs: Tumor-derived extracellular vesicles.

Changes in certain specific amino acids have been specifically attributed to one cancer type, facilitating its use as a diagnostic tool. For example, increased lysine has been detected in S-EVs from *in vitro* cells and serum from patients with colorectal cancer^[69]^, as well as in a heterogeneous population of EVs in stool^[75]^. Although changes in amino acid composition in stool EVs may be caused by microbiota dysregulation, the detection of increased lysine in S-EVs from serum and culture supernatants strongly supports the specificity of this marker for colorectal cancer cells. On the other hand, leucine and phenylalanine were upregulated in the L-EVs of pleural fluid samples from patients with cancer-related malignancies compared with those from patients with tuberculosis. Increased levels of these two amino acids were validated in two independent cohorts of patients as biomarkers to distinguish both malignancies^[93]^.

Other amino acids have been reported to be commonly modulated in EVs from cancers with no histological or mechanistic relationship. Indeed, glycine, valine and tryptophan are enriched in EVs isolated by ultracentrifugation from samples from colorectal cancer patients compared with those from healthy individuals^[75]^. On the other hand, the concentrations of glycine and tryptophan are increased in S-EVs released by glioblastoma cells with respect to their cytoplasmic abundance^[91]^, although not compared with those in noncancerous EVs. Moreover, Palviainen *et al.* demonstrated that proline was elevated in a heterogeneous population of EVs from prostate cancer, cutaneous T-cell lymphoma, and colon cancer as compared with noncancerous cell lines^[92]^. In contrast, the proline concentration was diminished in EVs isolated by ultracentrifugation from colorectal cancer patients in the study mentioned above^[75]^.

### Other metabolites

Since nucleotide synthesis is a crucial pathway sustaining metabolic reprogramming in cancer cells, detecting many nucleotides in TDEVs is common [Table 4]. Zhu *et al*. reported that 3’-uridine monophosphate (3’-UMP) is the most important biomarker in plasma S-EVs for distinguishing between the recurrent and nonrecurrent groups in esophageal squamous cell carcinoma^[81]^. Similarly, Hayasaka *et al*. reported that inosine, uridine, and cytidine are among the top 20 metabolites in S-EVs from pancreatic cancer cells^[95]^. In addition, Liu *et al*. reported low levels of dihydrothymidine in urine S-EVs derived from patients with prostate cancer^[99]^. Additionally, a panel of metabolites reflecting the upregulation of purine and pyrimidine synthesis in cancer cells found in urinary S-EVs (Kanzonol Z, Xanthosine, Nervonyl carnitine and 3,4-Dihydroxybenzaldehyde) differentiated lung cancer patients from healthy controls for early detection^[100]^.

**Table 4:** Other metabolites detected in TDEVs

**Class**	**Cancer type**	**Sample type**	**Metabolites**	**Application**	**Ref.**
Nucleotides	Oesophageal squamous cell carcinoma	Plasma recurrent and nonrecurrent	↑ 3’-UMP	Diagnosis	[81]
	Pancreatic cancer	Supernatant cells	↑ Inosine, uridine and cytidine	Diagnosis	[95]
	Prostate cancer	Urine	↓ Dihydrothymidine	Diagnosis	[96]
Organic acids	Colorectal cancer	Feces	↑ Furoic, succinic, oxalic	Diagnosis	[75]
	Head and neck cancer	Plasma	↑ Succinic acid ↓ Citric acid	Diagnosis	[97]
	Oesophageal squamous cell carcinoma	Plasma recurrent and nonrecurrent	↑ Organic acids and derivatives	Diagnosis	[81]
Other	Colorectal cancer	Feces	↑ Ethanolamine, phenol	Diagnosis	[75]
		Serum	↑ 1,4-dithiothreitol	Prognosis	[98]
	Endometrioid adenocarcinoma	Plasma	↑ Coenzyme Q10, ubiquinone 9, vitamin D3 derivatives, 10-formyldihydrofolate, acetylglucosamine bisphosphate, malonyl-CoA, picolinic acid	Diagnosis	[84]

The arrows indicate an increase (↑) or decrease (↓) in the concentration. TDEVs: Tumor-derived extracellular vesicles; 3’-UMP: 3’-uridine monophosphate.

The category of organic acids encompasses intermediate metabolites of critical metabolic pathways such as glycolysis and the tricarboxylic acid cycle (TCA), which are also present in EVs^[101]^. Zhu *et al*. reported that organic acids and their derivatives are abundant in plasma S-EVs from esophageal squamous cell carcinoma patients^[81]^. Moreover, succinic acid has been found to be increased, whereas citric acid is decreased in plasma S-EVs from patients with head and neck cancer^[97]^. Finally, furoic, succinic and oxalic acids are increased in EVs isolated by ultracentrifugation derived from colorectal cancer feces compared with healthy controls^[75]^.

Additional compounds have been detected in TDEVs. Kim *et al.* reported that alcohol-derived metabolites (ethanolamine and phenol) in fecal EVs were higher in patients with colorectal cancer than in healthy controls^[75]^. On the other hand, Strybel *et al.* revealed that 1,4-dithiothreitol in serum S-EVs is good at discriminating colorectal cancer patients with different responses to neoradiotherapy^[98]^. Finally, Altadill *et al.* identified several interesting metabolites in plasma EVs (containing both S and L-EVs) of endometrioid adenocarcinoma, such as coenzyme Q10, ubiquinone 9, 25-hydroxyhexadehydrovitamin D3, 10-formyldihydrofolate, acetylglucosamine bisphosphate, malonyl-CoA, picolinic acid and deoxyvitamin D3^[84]^.

In contrast, high cellular demand for glucuronate, D-ribose 5-phosphate and isobutyryl-L-carnitine leads to a reduction in these metabolites in urinary and platelet S-EVs from prostate cancer patients^[90]^. In this case, however, it should be noted that the changes were detected only from EVs by normalization to EV-derived factors or with metabolite ratios rather than from the original urine samples.

## METABOLITE CARGO IN THE CLINICAL SETTING: CHALLENGES

The detection of metabolites in TDEVs poses several challenges that must be carefully considered. First, and contrary to expectations, EVs are metabolically active units, indicating that, in some instances, the metabolite cargo does not necessarily reflect the content present in the producing cells^[102]^. Indeed, Iraci *et al*. reported that EVs (both S- and L-EVs) purified from neural stem/progenitor cells (NSCs) alter the levels of glutamate, GABA, aspartate and asparagine before they reach the cells, as they carry asparaginase with active enzymatic activity^[103]^. Another example is the production of ATP via functional ATP-forming enzymes in a heterogeneous population of prostate-derived EVs isolated by ultracentrifugation^[104]^.

Importantly, the metabolic state is naturally plastic and dynamic; thus, cellular and external signals such as inflammation or different stress sources can alter the cargo of TDEVs and even regulate their release process. Possible examples are the change in the lipid composition of the S-EV membrane caused by low pH or the increase in S-EV secretion under hypoxia induced by pyruvate kinase M2 (PKM2), an enzyme crucial for glucose metabolism in cancer cells^[105,106]^. Relatedly, the analysis of hydrophilic metabolites in the S-EVs of pancreatic cells under hypoxia revealed an increase in the levels of 2-deoxyribose 1-phosphate, a metabolite that is usually related to the adaptation of cell proliferation under hypoxia and resistance to hypoxic stress-induced apoptosis, as well as the promotion of angiogenesis^[95]^. Furthermore, the content of EVs isolated by ultracentrifugation can vary in pancreatic cancer after chemotherapeutic treatment, with an increase in the amino acid derivatives alanyl-histidine, 6-dimethylaminopurine, leucyl-proline and methionine sulfoxide^[94]^.

Moreover, it is crucial to consider different technical challenges. Indeed, several groups have reported that the metabolite cargo depends on the techniques used for EV production and isolation^[107,108]^. For example, the contents of both polar and nonpolar metabolites in S-EVs obtained from bioreactor-cultured cells differ significantly from those obtained from conventional cell culture^[109]^.

Finally, considering that the cargo can be considered a fingerprint from the producing cell, differences in metabolite content can reflect interpatient heterogeneity: S-EVs from the LIM125 cell line have higher levels of cholesterol, sphingolipids, glycerol and glycerolipids than those from other colorectal cancer cell lines^[74]^.

## METABOLISM-RELATED FUNCTIONS OF TDEVs

Cancer cells can influence the metabolism of noncancer cells present in the tumor microenvironment or even at distant sites via different mechanisms, to counteract nutrient scarcity and promote several of the cancer hallmarks mentioned in section 2 of this review. Considering that EVs transport genetic material, proteins, enzymes and metabolites within the tumor microenvironment, it is not surprising that they play a central role in promoting metabolic reprogramming in recipient cells^[110]^.

First, TDEVs can mediate metabolic cooperation between cancer cells and stromal cells, regulating nutrient availability. Indeed, cancer cells induce aerobic glycolysis in stromal cells and use their end products, such as pyruvate and lactate, to fuel mitochondrial oxidative phosphorylation through the reverse Warburg effect^[111]^. Since both the Warburg effect and the reverse Warburg effect share the same key enzymes, the latter process can be mediated by TDEVs through the transport of glycolytic enzymes, which are among the 100 proteins most identified in TDEVs^[112]^. For example, epithelial cancer cells promote glycolysis in CAFs via the transfer of PKM2 and LDH^[113]^. In addition to direct enzyme transfer, an alternative mechanism for reverse Warburg induction is mediated by nucleic acids. For example, Yan *et al.* reported that breast cancer cells release a mixed population of EVs containing miR-105 that induce metabolic reprogramming in CAFs in a process mediated by the oncoprotein c-MYC^[114]^. In this report, the authors describe a model in which CAFs sense metabolic needs in the tumor microenvironment, modulating their metabolism in order to maintain cancer cell proliferation. Similarly, S-EVs derived from CAFs in prostate and pancreatic cancer have been found to be enriched in essential amino acids such as glutamine and arginine, which are used as nutrients by cancer cells. In addition, they can increase glutamine-dependent reductive carboxylation when they enter cancer cells^[115,116]^.

In addition to CAFs, TDEVs have also been shown to mediate metabolic reprogramming in other stromal cells present in the tumor microenvironment or even in distant tissues, thus promoting systemic changes. For example, TDEVs from Lewis lung cancer cells exhibit increased levels of phosphorylated hormone-sensitive lipase (p-HSL), an enzyme that mediates lipolysis. Indeed, incubation of 3T3-L1 adipocytes with EVs isolated by ultracentrifugation induced lipolysis, which was detected as a lower content of lipid droplets and increased glycerol release. Interestingly, these findings suggest that TDEVs may contribute to systemic cachexia by inducing adipocyte browning^[117]^. On the other hand, breast tumor cells secrete a mixed population of EVs with high levels of miR-122 to inhibit glucose uptake by nontumor cells in distant premetastatic areas, thus facilitating metastasis by increasing glucose bioavailability^[118]^.

In addition to metabolic cooperation, TDVEs facilitate tumor progression via metabolic reprogramming of stromal cells, resulting in angiogenesis or immunoevasion. For example, Wang *et al*. demonstrated that S-EVs secreted by acute myeloid leukemia cells contain VEGF and VEGFR mRNAs, which promote VEGFR expression and subsequent glycolysis in endothelial cells, thus causing vascular remodeling and the acquisition of chemoresistance^[119]^. Interestingly, chemokines and miRNAs transported in S-EVs increase oxidative phosphorylation concomitant with glycolysis inhibition in macrophages, promoting their differentiation into tumor-associated macrophages with immunoevasive properties^[120]^. Furthermore, EVs isolated by ultracentrifugation and secreted by pancreatic ductal adenocarcinoma cells with SMAD4 expression expand myeloid-derived suppressor cells by inducing calcium flux and glycolysis, thus promoting an immunosuppressive background^[121]^.

## CONCLUSION

**Figure 1:**
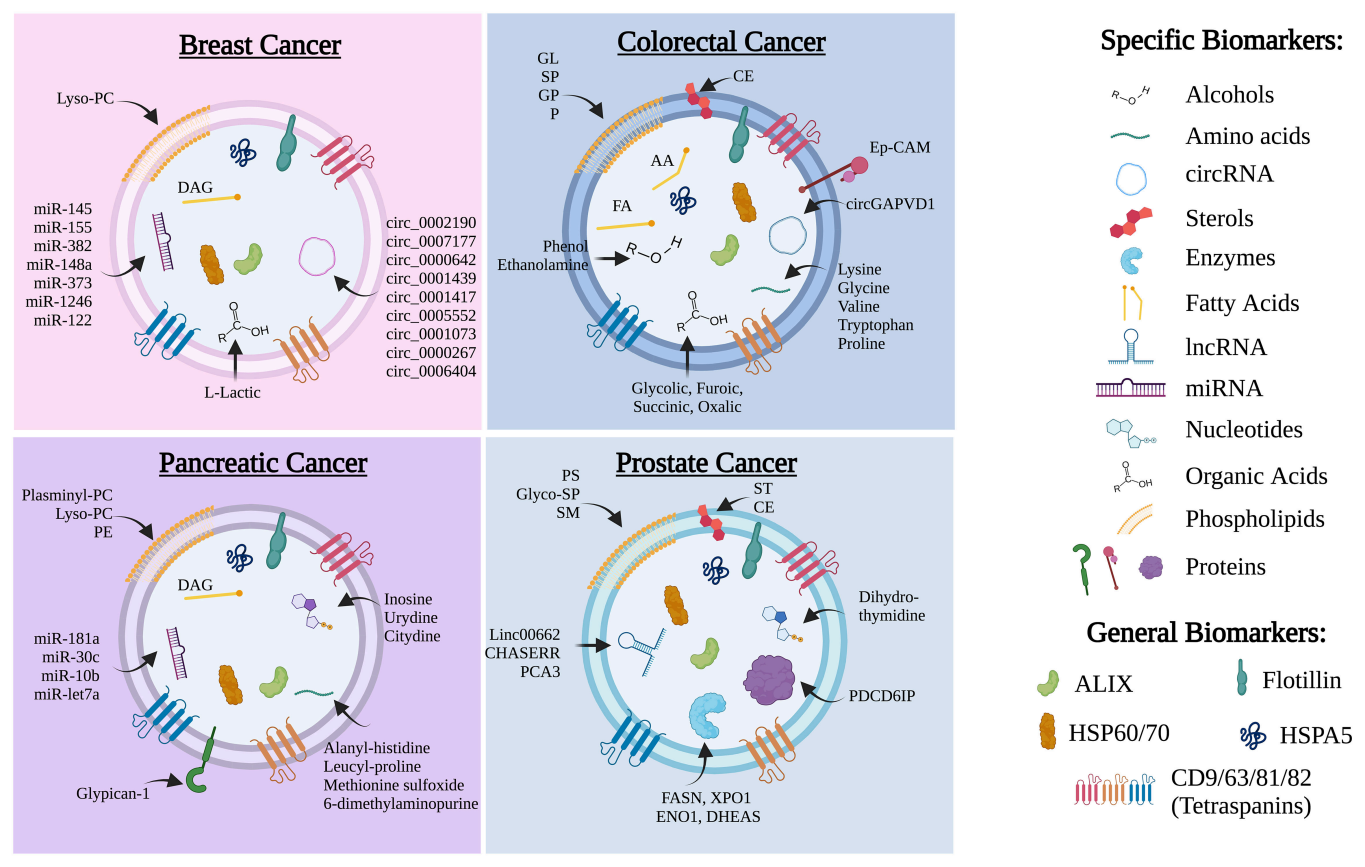
Biomarkers of TDEVs described in four representative cancer types. Specific biomarkers (proteins, nucleic acids and metabolites) as well as general EV-related biomarkers have been included for breast, colorectal, pancreatic, and prostate cancers. Lipids: CE: cholesteryl esters; DAG: diacylglycerol; FA: fatty acids; GL: glycolipids; GP: glycerophospholipids. Glyco-PS: glycophosphosphingolipids; Lyso-PC: lysophospatidylcholine; P: propionate; PC: phosphatidylcholine; PE: phosphatidyletanolamine; PS: phosphatidylserine; SM: sphingomyelin; SP: sphingolipids; ST: sterols; TDEVs: tumor-derived extracellular vesicles.

Over the past few years, the crucial role of TDEVs in cancer initiation and progression has become increasingly clear. Indeed, they contribute to the bidirectional communication between cancer cells and stromal cells in both local and distant microenvironments, which promotes phenotypic changes involving metabolic reprogramming, angiogenesis, drug resistance, metastasis and immunosuppression, among other processes. In this sense, the increased secretion of EVs by cancer cells and the detection of TDEVs in a variety of biofluids have attracted the attention of the scientific community to the study of these vesicles for diagnostic and prognostic purposes, as well as potential targets for cancer treatment. TDEVs carry specific cargoes composed of nucleic acids, proteins, and metabolites, all of which have potential bioactivity (examples in four specific cancer types are summarized in Figure 1). For example, metabolites contained in TDEVs can reflect the metabolic state of the producing cells, although we need to consider that cellular metabolism is plastic and dynamic depending on the cellular context, microenvironmental conditions, and other factors. This metabolite cargo may have various effects on receptor cells, for example, providing key nutrients to support the proliferation of cancer cells or inducing metabolic reprogramming to promote a system of metabolic cooperation between cancer cells and stromal cells. However, in addition to nutrient interchange, very little is known about the mechanisms mediating the effects of this metabolic cargo on receptor cells and the duration of these effects.

The field of EV metabolism is actively evolving, but important challenges need to be addressed. First, the variety of biological samples used (supernatants from cell lines cultured under different conditions, and patient samples from different origins) is the main reason for the lack of consistency among studies found in the literature. Undeniably, studies performed in patient samples are especially relevant, but most of these studies include few samples that were collected at a specific center. In this sense, it would be essential to perform studies in large multicentric cohorts with standardized protocols for sampling and subsequent processing. Indeed, technical problems derived from isolation and purification methodologies can largely affect the EV population analyzed (S or L-EVs or a mixture of both), their content, and cross-contamination from normal *vs.* TDEVs. Additionally, the techniques used to reliably detect metabolites in the low concentration ranges found in TDEVs are still evolving.

In summary, although it holds great potential, the field of EV metabolism in cancer is still in its infancy, and a joint effort by the scientific community on methodology standardization is essential to advance.

## DECLARATIONS

### Copyright

© The Author(s) 2024.
