# Supplementary material for: Metabolic features of tumor-derived extracellular vesicles: challenges and opportunities
Source: Extracell Vesicles Circ Nucl Acids. 2024 Aug 27;5(3):455–70. doi: 10.20517/evcna.2024.12 (PMC11648520; doi:10.20517/evcna.2024.12)
Supplement: Supplementary file 1 [file evcna-5-3-455-SupplementaryMaterials.pdf]

Espiau-Romera *et al. Extracell Vesicles Circ Nucleic Acids* 2024;5:555-70
DOI: 10.20517/evcna.2024.12

Extracellular Vesicles and Circulating Nucleic Acids

Supplementary Materials

Metabolic features of tumor-derived extracellular vesicles: challenges and opportunities

Pilar Espiau-Romera, Andrés Gordo-Ortiz, Inés Ortiz-de-Solórzano, Patricia Sancho

Hospital Universitario Miguel Servet, IIS Aragón, Zaragoza 50009, Spain.

Correspondence to: Dr. Patricia Sancho, Hospital Universitario Miguel Servet, IIS Aragón, Av. Isabel la Católica 1-3, Zaragoza 50009, Spain. E-mail: psancho@iisaragon.es

Supplementary Table 1. Examples of general EVs markers used for TDEV isolation in different sample types

Marker	Source of EVs	Cancer Type	Ref.
ALIX	Plasma	Pancreatic cancer	[1]
CCT2 (HSP)	Blood	Breast cancer	[2]
CD63	Plasma	Melanoma	[3]
CD81	Lung cancer cell line	Non-Small cell lung cancer	[4]
CD82	Serum	Breast cancer	[5]
CD9	Urine	Prostate cancer	[6]
Flotillin	Urine	Prostate cancer	[7]
HSP60	Cell lines (NCI-H292, A549, K562)	Lung cancer and leukaemia	[8]
HSP70	Human pancreas and colorectal carcinoma cells	Pancreatic and colorectal cancer	[9]
HSPA5	Cell lines (K562, U87-MG, SW480)	Leukaemia, glioblastoma and colorectal cancer	[10]
TSG101	Colorectal carcinoma tissue	Colorectal cancer	[11]

Supplementary Table 2. Nucleic acids in TDEVs

Nucleic acid	Biomarker	Source of EVs	Cancer Specificity	Application	Ref.
circRNA	circ_0002190	Plasma	Breast cancer	Diagnosis	[12]
	circ_0007177				
	circ_0000642				
	circ_0001439				
	circ_0001417				
	circ_0005552				
	circ_0001073				
	circ_0000267				
	circ_0006404				
	circGAPVD1	Plasma	Colorectal cancer	Diagnosis	[13]
	circNRIP1	Plasma	Gastric cancer	Early diagnosis	[14]
	circ_0051443	Plasma	Hepatocellular carcinoma	Treatment response	[15]
lncRNA	Linc00662	Urine	Prostate cancer	Early diagnosis	[16]
	CHASERR				
	PCA3				
miRNA	miR-145	Serum	Breast cancer	Early diagnosis	[17]
	miR-155				
	miR-382				
	miR-148a				
	miR-373			Prognosis	[18]
	miR-1246			Diagnosis	[19]
	miR-122			Treatment response	[19]
	miR-21-5p	Serum	Hepatocellular carcinoma	Early diagnosis	[20]

	miR-378a	Serum	Lung cancer	Diagnosis	[21]
	miR-379				
	miR-139-5p				
	miR-200b-5p				
	miR-151a-5p				
	miR-30a-3p				
	miR-629				
	miR-100				
	miR-154-3p				
	miR-181a	Serum	Pancreatic ductal adenocarcinoma	Diagnosis	[22]
	let-7a family				
	miR-10b				
	miR-30c				
tRNA	tRNA-ValAAC-5	Plasma	Liver cancer	Diagnosis	[23]
	tRNA-GluCTC-5				
	tRNA-ValTAC-3			Early diagnosis	
	tRNA-GlyTCC-5				
	tRF-Leu-TAA-005	Plasma	Non-small cell lung cancer	Diagnosis	[24]
	tRF-Asn-GTT-010				
	tRF-Ala-AGC-036				
	tRF-Lys-CTT-049				
	tRF-Trp-CCA-057				

References

1. Yang J, Zhang Y, Gao X, *et al.* Plasma-Derived Exosomal ALIX as a Novel Biomarker for Diagnosis and Classification of Pancreatic Cancer. *Front Oncol* 2021;11:628346. [DOI: 10.3389/fonc.2021.628346].
2. Alagundagi DB, Ghate SD, Rajendra VKJ, *et al.* Exploring breast cancer exosomes for novel biomarkers of potential diagnostic and prognostic importance. *3 Biotech* 2023;13:7. [DOI: 10.1007/s13205-022-03422-w].
3. Logozzi M, De Milito A, Lugini L, *et al.* High levels of exosomes expressing CD63 and caveolin-1 in plasma of melanoma patients. *PLoS One* 2009;4:e5219. [PMID: 19381331 DOI: 10.1371/journal.pone.0005219].
4. Signorelli D, Ghidotti P, Proto C, *et al.* Circulating CD81-expressing extracellular vesicles as biomarkers of response for immune-checkpoint inhibitors in advanced NSCLC. *Front Immunol* 2022;13:987639. [DOI: 10.3389/fimmu.2022.987639].
5. Wang X, Zhong W, Bu J, *et al.* Exosomal protein CD82 as a diagnostic biomarker for precision medicine for breast cancer. *Molecular Carcinogenesis* 2019;58:674–85. [DOI: 10.1002/mc.22960].
6. Soekmadji C, Riches JD, Russell PJ, *et al.* Modulation of paracrine signaling by CD9 positive small extracellular vesicles mediates cellular growth of androgen deprived prostate cancer. *Oncotarget* 2017;8:52237–55. [DOI: 10.18632/oncotarget.11111].
7. Wang L, Skotland T, Berge V, Sandvig K, Llorente A. Exosomal proteins as prostate cancer biomarkers in urine: From mass spectrometry discovery to immunoassay-based validation. *European Journal of Pharmaceutical Sciences* 2017;98:80–5. [DOI: 10.1016/j.ejps.2016.09.023].
8. Merendino AM, Bucchieri F, Campanella C, *et al.* Hsp60 Is Actively Secreted by Human Tumor Cells. Tailleux L, editor. *PLoS ONE* 2010;5:e9247. [DOI: 10.1371/journal.pone.0009247].
9. Gastpar R, Gehrmann M, Bausero MA, *et al.* Heat Shock Protein 70 Surface-Positive Tumor Exosomes Stimulate Migratory and Cytolytic Activity of Natural Killer Cells. *Cancer Research* 2005;65:5238–47. [DOI: 10.1158/0008-5472.CAN-04-3804].
10. Wang Q, Yu C. Identification of biomarkers associated with extracellular

vesicles based on an integrative pan-cancer bioinformatics analysis. *Med Oncol* 2020;37:79. [DOI: 10.1007/s12032-020-01404-7].

11. Gheytanchi E, Saeednejad Zanjani L, Ghods R, *et al.* High expression of tumor susceptibility gene 101 (TSG101) is associated with more aggressive behavior in colorectal carcinoma. *J Cancer Res Clin Oncol* 2021;147:1631–46. [DOI: 10.1007/s00432-021-03561-2].

12. Lin L, Cai G-X, Zhai X-M, *et al.* Plasma-Derived Extracellular Vesicles Circular RNAs Serve as Biomarkers for Breast Cancer Diagnosis. *Front Oncol* 2021;11:752651. [DOI: 10.3389/fonc.2021.752651].

13. Li T, Zhou T, Wu J, *et al.* Plasma exosome-derived circGAPVD1 as a potential diagnostic marker for colorectal cancer. *Translational Oncology* 2023;31:101652. [DOI: 10.1016/j.tranon.2023.101652].

14. Zhang X, Wang S, Wang H, *et al.* Circular RNA circNRIP1 acts as a microRNA-149-5p sponge to promote gastric cancer progression via the AKT1/mTOR pathway. *Mol Cancer* 2019;18:20. [DOI: 10.1186/s12943-018-0935-5].

15. Chen W, Quan Y, Fan S, *et al.* Exosome-transmitted circular RNA hsa_circ_0051443 suppresses hepatocellular carcinoma progression. *Cancer Letters* 2020;475:119–28. [DOI: 10.1016/j.canlet.2020.01.022].

16. Bajo-Santos C, Brokāne A, Zayakin P, *et al.* Plasma and urinary extracellular vesicles as a source of RNA biomarkers for prostate cancer in liquid biopsies. *Front Mol Biosci* 2023;10:980433. [DOI: 10.3389/fmolb.2023.980433].

17. Gonzalez-Villasana V, Rashed MH, Gonzalez-Cantú Y, *et al.* Presence of Circulating miR-145, miR-155, and miR-382 in Exosomes Isolated from Serum of Breast Cancer Patients and Healthy Donors. *Disease Markers* 2019;2019:1–9. [DOI: 10.1155/2019/6852917].

18. Eichelser C, Stückrath I, Müller V, *et al.* Increased serum levels of circulating exosomal microRNA-373 in receptor-negative breast cancer patients. *Oncotarget* 2014;5:9650–63. [DOI: 10.18632/oncotarget.2520].

19. Hannafon BN, Trigoso YD, Calloway CL, *et al.* Plasma exosome microRNAs are indicative of breast cancer. *Breast Cancer Res* 2016;18:90. [DOI: 10.1186/s13058-016-0753-x].

20. Ghosh S, Bhowmik S, Majumdar S, *et al.* The exosome encapsulated MICRORNAS as circulating diagnostic marker for hepatocellular carcinoma with low alpha-fetoprotein. *Intl Journal of Cancer* 2020;147:2934–47. [DOI:

10.1002/ijc.33111].
21. Cazzoli R, Buttitta F, Di Nicola M, *et al.* microRNAs Derived from Circulating Exosomes as Noninvasive Biomarkers for Screening and Diagnosing Lung Cancer. *Journal of Thoracic Oncology* 2013;8:1156–62. [DOI: 10.1097/JTO.0b013e318299ac32].
22. Lai X, Wang M, McElyea SD, Sherman S, House M, Korc M. A microRNA signature in circulating exosomes is superior to exosomal glypican-1 levels for diagnosing pancreatic cancer. *Cancer Letters* 2017;393:86–93. [DOI: 10.1016/j.canlet.2017.02.019].
23. Zhu L, Li J, Gong Y, *et al.* Exosomal tRNA-derived small RNA as a promising biomarker for cancer diagnosis. *Mol Cancer* 2019;18:74. [DOI: 10.1186/s12943-019-1000-8].
24. Zheng B, Song X, Wang L, *et al.* Plasma exosomal tRNA-derived fragments as diagnostic biomarkers in non-small cell lung cancer. *Front Oncol* 2022;12:1037523. [DOI: 10.3389/fonc.2022.1037523].
